# Continuity of care and multimorbidity in the 50+ Swiss population: An analysis of claims data

**DOI:** 10.1016/j.ssmph.2022.101063

**Published:** 2022-03-09

**Authors:** Anna Nicolet, Isabelle Peytremann-Bridevaux, Christophe Bagnoud, Clémence Perraudin, Joël Wagner, Joachim Marti

**Affiliations:** a Center for Primary Care and Public Health (Unisanté), University of Lausanne, Lausanne, Switzerland; b Groupe Mutuel, Rue des Cèdres 5, Case Postale, CH-1919, Martigny, Switzerland; c Department of Actuarial Science, Faculty of Business and Economics (HEC), And Swiss Finance Institute, University of Lausanne, Lausanne, Switzerland

**Keywords:** Care continuity, Multimorbidity, Claims data, Pharmacy-cost groups, Association

## Abstract

**Objective:**

To assess the relationship between continuity of care (COC) and multimorbidity in the older general population in Switzerland, accounting for relevant determinants of COC, and to apply various expressions of multimorbidity derived from claims data.

**Methods:**

We used data on 240′000 insured individuals aged 50+ for the period 2015–2018, received from one of the largest Swiss health insurance company. We calculated Bice-Boxerman index based on all doctor visits (overall COC) and visits to the general practitioners (COC GP). We analyzed the relationship between COC and multimorbidity using generalized linear and probit models. To express multimorbidity, we applied three approaches based on pharmacy-cost groups (PCGs) assigned to an individual. First, we used simple PCG counts. Second, we expressed multimorbidity via clinically relevant disease groups derived from PCGs. Finally, a data-driven approach allowed defining distinct clusters representing different patient complexities.

**Results:**

The association between overall COC and multimorbidity expressed in PCG counts was modest: COC among individuals with 3+ PCGs was 2 percentage points higher than COC among individuals with 0 PCGs. The approach of clinically relevant disease groups showed larger variation in COC and its association with multimorbidity. The data-driven approach showed that most complex (“high-cost high-need”) individuals tended to have higher overall COC. Additionally, 70% of the sample visited exclusively one general practitioner (COC GP = 1.0). Other important factors associated with COC in the Swiss context were insurance model with gatekeeping, level of deductibles, and region of residence.

**Conclusions:**

Multimorbid patients require regular medical attention often involving multiple healthcare providers, which can lead to varying COC, depending on types of doctors seen and specific condition of the patient. Insurance models with gatekeeping may facilitate COC, prompting developments of better-designed models of care. This represents important implications for policymakers, health insurance representatives, medical professionals and hospital managers.

## Highlights

• We investigated the relationship between multimorbidity and COC, using claims-based data.
• We applied three approaches of expressing multimorbidity in claims data: simple counts, expert-based and data-driven.
• Association between COC and multimorbidity expressed in simple counts was modest, while expert-based approach showed larger heterogeneity.
• Data-driven approach revealed that most complex individuals tended to have higher COC.

## 1 Introduction

The increasing number of people affected by multiple chronic diseases poses considerable challenges for healthcare systems worldwide in terms of care organization, planning, and consequently costs (Fortin, Soubhi, Hudon, Bayliss, & van den Akker, 2007; Onder et al., 2015). Multimorbid patients are often characterized by having high healthcare utilization, complex needs, multiple healthcare providers and medications, and frequent transitions between care settings (Prince et al., 2015). Ensuring smooth transitions between various care settings with multiple providers, while considering patient needs, is currently not universally achieved. One of the reasons is the traditional design of healthcare systems and clinical practice guidelines, structured around pathways of care for single diseases, which can have undesirable effects and potentially harmful implications stemming from highly fragmented care (Muth et al., 2019; Onder et al., 2015; Vetrano et al., 2018). As a result of high fragmentation and lack of sustainable relationships between patients and health professionals, patients with multiple chronic diseases are particularly at risk of unnecessary repeated diagnostic tests, adverse drug interactions, and may have lower quality of life (Boyd et al., 2005; Larsen, Pedersen, Friis, Glumer, & Lasgaard, 2017; N'Goran et al., 2017). To improve healthcare delivery for such patients, a shift is needed towards more structured and coordinated care delivery, with enhanced sustainable professional collaboration and greater support for patients.

In 2014, Hussey and colleagues highlighted continuity of care (COC) as a distinct and eminently measurable component of care coordination (Hussey et al., 2014). COC reflects regular visits to a health professional, sustained over time; a relationship of trust and responsibility between patients and health professionals (Meiqari, Al-Oudat, Essink, Scheele, & Wright, 2019). In fact, COC has two core elements (Haggerty et al., 2003): care of an individual patient and care over time, distinguishing it from care coordination and integration (Rijken et al., 2018). Research has shown that poor COC is associated with not only higher health care costs and more hospitalizations (Bazemore et al., 2018; CDKnight, Dowden, Worrall, Gadag, & Murphy, 2009; Chen, Yamada, Smith, & Chiu, 2011; Cho et al., 2015, Cho et al., 2015; Chu, Chen, & Cheng, 2012; Hong & Kang, 2013; Hong, Kang, & Kim, 2010; Jung, Cho, Lee, & Kim, 2018; Kao & Wu, 2016, 2017; Lin & Wu, 2017; Pollack, Weissman, Lemke, Hussey, & Weiner, 2013; Romaire, Haber, Wensky, & McCall, 2014), but with worse health outcomes, especially in chronic and multimorbid patients (Cho, Kim, et al., 2015; Gruneir et al., 2016; Jang, Choy, Nam, Moon, & Park, 2018; Weir, McAlister, Majumdar, & Eurich, 2016; Ye et al., 2016). However, the meaning behind COC can vary depending on the used definitions (Gulliford, Naithani, & Morgan, 2006; Haggerty et al., 2003; Saultz, 2003; Saultz & Albedaiwi, 2004; Shortell, 1976), COC measurement methods and data sources.

In Switzerland, with its highly decentralized healthcare system, characterized by a complex division of responsibilities and financing mechanisms, maintaining structured coordinated care and reducing care fragmentation for multimorbid patients are important challenges. Fragmentation of care trajectories may be potentially associated with poor coordination, duplication of low-value services, or polypharmacy (Schussele Filliettaz, Berchtold, Kohler, & Peytremann-Bridevaux, 2018). In fact, poor COC was found to be negatively associated with a range of patient outcomes affecting particularly patients with multiple chronic diseases since several healthcare providers are usually involved in their care (Gruneir et al., 2016; Hussey et al., 2014; Van Walraven, Oake, Jennings, & Forster, 2010; Wang et al., 2020; Weir et al., 2016). Additional challenges for the Swiss health system are high share of GDP spent on healthcare (>12%) and high contribution of households, including insurance premiums and out-of-pocket payments (e.g., deductibles and co-payments) (De Pietro et al., 2015). Insurance premiums are community-rated and do not depend on income, and means-tested insurance subsidies exist to support lower-income households. Basic health insurance is compulsory in Switzerland, and Swiss residents can individually choose their insurer independently of their employer, and the insurers cannot reject applicants. As of 2022, there are 51 health insurance companies offering different models with several possible levels of annual deductibles (from 300 CHF to 2500 CHF), which creates a challenge for many individuals to make the optimal choice. Costs beyond the annual deductible are paid by the insurer, but the individuals still have to cover 10% of remaining costs up to 700 Swiss francs annually (co-payments). Although basic insurance allows freedom of access to specialists and unlimited access to general practitioners, alternative models with restricted choice of providers grant discounts in monthly premiums. Such models include access conditional on GP referral (i.e. gatekeeping), or access to a limited set of selectively contracted providers (De Pietro et al., 2015). Thus, in fragmented health systems with free provider choice, lack of care coordination and primary care weaknesses such as Switzerland (Schussele Filliettaz et al., 2018), COC becomes of particular importance for older adults with multiple chronic conditions. The impact of multimorbidity on costs and health outcomes in Switzerland has been investigated (Bahler, Huber, Brungger, & Reich, 2015), but the relationship between multimorbidity and COC remains undocumented. In Switzerland there is no global database or registry of patient medical information, therefore, studies increasingly rely on retrospective claims-based data. Such data are mainly collected for billing and reimbursement purposes, ensuring their regular and comprehensive collection and management, but are lacking details on diagnoses. Often, multimorbidity measures rely on morbidity indices (e.g, Charlson, Elixhauser) or on the number of (self-reported) chronic conditions or co-morbidities (Sharabiani, Aylin, & Bottle, 2012). The former were developed in an inpatient setting as predictors of mortality, and the latter may not comprehensively reflect the patient's disease burden and complexity. In countries without global registries, like Switzerland, where clinical information is not universally available, the application of standard methods for measuring multimorbidity is not feasible. Researchers may therefore rely on an ad-hoc approach that has been developed specifically for Swiss claims data (Chini, Pezzotti, Orzella, Borgia, & Guasticchi, 2011; Huber, Szucs, Rapold, & Reich, 2013).

The main aim of our descriptive study was to investigate the relationship between continuity of care (visit-based COC) and multimorbidity in the older general population in Switzerland, taking into account the determinants of COC relevant in the Swiss context, and to apply various expressions of multimorbidity derived from claims data.

## 2 Methods

### 2.1 Data source, study design and sample

Our descriptive observational study was based on retrospective analysis of claims data, obtained from Groupe Mutuel, one of the largest health insurance companies in Switzerland. According to the recent statistics of 2019, it covered 982′379 individuals with mandatory health insurance, representing 11.4% of the insured individuals in Switzerland ((BAG), 2019). The dataset includes information on more than 240′000 continuously enrolled individuals aged 50+ years (70% of all insured aged 50+, randomly selected by Groupe Mutuel) in all Swiss cantons with higher representation of the French-speaking region (i.e. Jura, Neuchatel, Geneva, Vaud, Valais, and Fribourg) and covers the 2015–2018 period. Besides basic demographic information (age, gender, region of residence), the data from Groupe Mutuel contains number of reimbursed visits to various physicians with associated specializations, information on the health insurance model with deductibles level, number of hospital admissions with the length of stay, costs of used medications, ambulatory and stationary costs.

### 2.2 Measuring COC. Outcome measure

We applied the Bice-Boxerman continuity of care index (COCI), the most commonly used index in the literature on COC (Hussey et al., 2014; Jee & Cabana, 2006; Van Walraven et al., 2010). This measure assigns a value between zero and one to each patient, with one indicating the highest possible concentration of doctor visits, and thus, the highest COC, and zero indicating absence of continuity whereby each time a different doctor is visited.

$$COCI=\frac{\sum_{i=1}^{M}n_{i}²-N}{N\left(N-1\right)}$$

*M* – total number of providers, *N* – total number of visits, *n*_*i*_ – number of visits to provider *i.*

For each subject we calculated two types of continuity measures. For the first type of measures, we determined the overall COCI index taking into account all contacts with physicians across all specialties covered by health insurance bills (“overall COCI”).^1^ The overall COCI is highly influenced by the diversity of the medical care a patient receives. For the second type of measures, we calculated COCI based only on visits to general practitioners (GPs), thus, measuring continuity of primary care with a general practitioner (“COCI GP”). Since COC is only meaningful when multiple visits to healthcare providers are undertaken, we calculated the COCI and used it in the analyses for individuals with three and more visits per year (N = 171′646 for COCI overall, and N = 133′929 for COCI GP in 2015).

### 2.3 Measuring multimorbidity

**Fig. 1:**
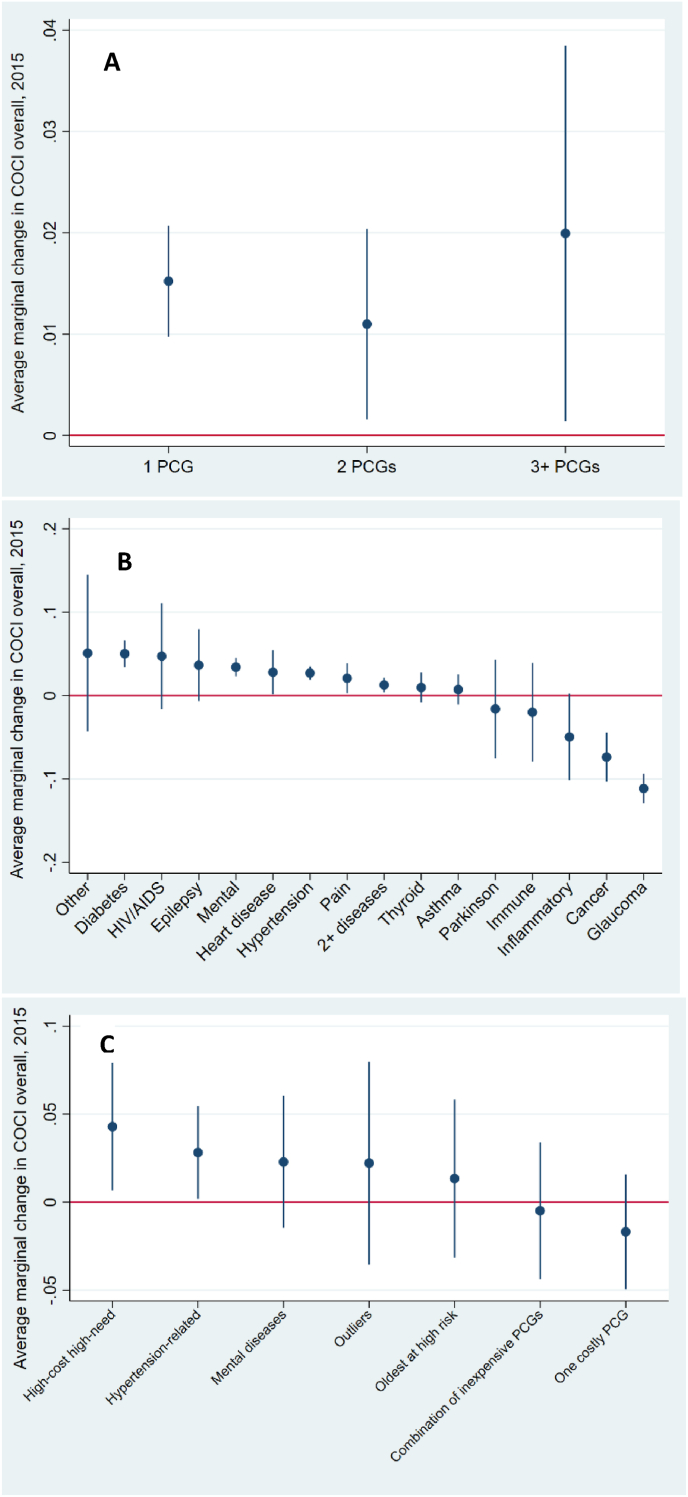
Average marginal effects in percentage points in 2015 of various multimorbidity specifications on overall COCI (fully adjusted model) **A)** simple PCG counts approach **B)** clinically relevant disease groups of PCGs (expert-based approach) **C)** cluster analysis (data-driven approach).

To identify insured persons with cost-intensive chronic diseases and correspondingly high healthcare utilization based on their drug consumption, health insurance companies are translating the drug utilization data reflecting active ingredient and quantity, based on ATC (Anatomical Therapeutic Chemical) and DDD (Defined Daily Dose), into pharmacy-cost groups (PCGs) (“Schlussbericht: Aktualisierung der PCG-Liste für den Schweizer Risikoausgleich,”). Enrollees were attributed individual PCGs, reflecting diseases according to the official PCG-disease mapping procedure developed and officially accepted by the Federal office of Public Health in Switzerland (“Schlussbericht: Aktualisierung der PCG-Liste für den Schweizer Risikoausgleich,”). Using this general assessment of the morbidity status, we considered three different ways of expressing multimorbidity. First, morbidity characterized by the number of PCGs attributed to each enrollee (“PCG counts”): 0 PCGs (i.e. no morbidities), 1 and 2 PCGs, 3+ PCGs – multimorbid, an approach suggested in an earlier study (Johnston, Crilly, Black, Prescott, & Mercer, 2019). Since simple counts may have limitations (Chini et al., 2011), we then decided to apply a more “clinical-based approach” and investigated whether having particular diseases (derived from PCGs) would reveal a different relationship with COC than a simple count. To do so, we involved two clinical experts to allocate 34 PCGs into disease groups, meaningful from a clinical perspective, which resulted in 17 disease groups (Supplement, Table 1 and Fig. 1). Taken into account the lack of individuals' diagnoses, we considered involving clinical experts for reducing the amount of groups from 34 to 17 as a feasible approach to investigate the potential effect of distinct diseases, beyond simple counts. A third “data-driven” way of expressing morbidity was based on previous cluster analysis where groups with homogenous patterns (clusters) were detected using classification and regression tree-based method including machine-learning algorithms (Breiman, 2001; Nicolet et al., 2022; Shi, Seligson, Belldegrun, Palotie, & Horvath, 2005). Being computationally expensive, the cluster analysis was performed only on 10% of the sample (*N* = 18′732), whereby the distribution of PCGs and background characteristics were preserved and remained similar to the full dataset. Individuals were allocated into one of the clusters, expressing similar patterns of healthcare use and costs, based on their PCG-based conditions. Apart from outliers, the identified clusters were “No morbidity” (no assigned PCGs, youngest, lowest healthcare use and costs), “High-cost high-need”, “Combination of inexpensive PCGs”, “Oldest persons at high risk”, “One costly PCG”, “Hypertension-related diseases only” (assigned only PCGs related to Hypertension diseases), and “Mental diseases only” (assigned PCGs related to Mental diseases only). The members of cluster “High-cost high-need patients” are characterized by the highest number of PCGs often appearing jointly, highest costs and healthcare use, and the members of cluster “Patients with combination of inexpensive PCGs” although having multiple PCGs (examples: Thyroid, Hypertension, Glaucoma and mix of others) appearing jointly less often, had healthcare costs and use lower than in previous cluster. The members of cluster “Oldest persons at high risk” had PCGs (Asthma, or Parkinson, or Cardiac diseases, or Pain) rarely appearing jointly, were of oldest age with especially high use of hospitalizations and visits to the generalist doctor, and high stationary costs. The members of cluster “One costly PCG” were characterized by a relatively small number of PCGs almost never appearing jointly (single diseases) and highest costs of medications.

### 2.4 Statistical analysis

First, we conducted descriptive uni- and bivariate analyses and present information on overall COCI in each subgroup of the (multi)morbidity expressions, alongside with its main components: total visits, visits to specialists and to GPs, and proportions of individuals visiting exclusively one GP.

Second, the association between overall COCI and morbidity was investigated using generalized linear models, estimated for each morbidity expression (GLM, family binomial, link logit, Stata software) for the baseline year 2015. All models included other potentially important background explanatory variables for associations estimation: age, gender, type of health insurance contract (with or without gatekeeping), region (French-speaking part versus other regions), and level of deductible (300 CHF, 500 CHF, over 500 CHF). As the COCI GP often attained the maximum value (1.0 meaning that participants visit exclusively one GP), we used a probit model, instead of generalized linear model, to estimate the probability of having visits exclusively to one GP (COCI GP = 1.0). Cluster analysis was performed on 10% of the sample, therefore, models for the associations between COC (overall and GP COCI) and cluster membership were performed on the same 10% individuals. Coefficients were transformed to average marginal effects for ease of interpretation. Average marginal effects show how, on average, a dependent variable (COCI in our case) changes when the levels of the explanatory variables change (or at a one-unit change of the explanatory variables). No attributed PCGs (“0 PCGs”) was the reference category in all models for all multimorbidity expression approaches.

While it is common practice to categorize measures of COC before the analysis, we decided to keep them continuous to avoid problems associated with the choice of arbitrary cut-points or the categorization of variability within each group (Geroldinger et al., 2018).

Ethics approval for this study was waived by The Cantonal Commission for the Ethics of Research on Human Beings (CER-VD, Lausanne, Switzerland).

## 3 Results

### 3.1 Sample characteristics

The sample consisted of 240′419 enrollees in 2015 (Supplement, Table 2). The mean age was 63.9 years, while almost 48% of the sample were males. Half of the individuals had insurance models with restricted access to the specialists (i.e., gatekeeping model) and a little less than half had the lowest possible level of deductibles (300 CHF). The number of individuals with at least one hospitalization was 13.2% in 2015, and the mean length of stay was 2.2 days. The total average number of doctor consultations was 10.0, while 5.6 consultations were with GPs. Most enrollees did not have any PCGs, but this proportion within enrollees decreased in 4 years from 72.0% to 63.0%. On the other hand, the proportion of enrollees with multiple PCGs rose from 6.6% in 2015 to 10.1% in 2018, reflecting likely health deterioration within sample with years. The mean number of different specialist doctors increased with (multi)morbidity status: 4.7 for those with 0 PCGs to 6.0 for those with 2+ PCGs in 2015. Mean overall COC (and COC GP) slightly decreased from 0.51 (0.89) in 2015, to 0.50 (0.88) in 2018 (Supplement, Table 2).

### 3.2 Associations between COC and (multi)morbidity expressed via PCG counts

With increasing PCGs, the overall number of doctor visits (specialists as well as GPs) increased: 7.9 average total visits for individuals with 0 PCGs to 23.5 for those with 3+ PCGs (Table 1a). In all PCG counts groups, individuals had more visits to GPs than to specialists.

**Table 1:** COC and visit patterns in a year of 2015, by various morbidity specifications*.

Table 1a	Simple counts of PCGs			
	0	1	2	3+
Overall visits (to specialists; to GPs)	7.9 (3.5; 4.5)	14.0 (6.1; 7.9)	18.7 (8.6; 10.1)	23.5 (10.7; 12.7)
% individuals with one GP (COCI GP = 1)	73.8	73.5	71.2	66.1
Mean overall COCI (IQR)	0.51 (0.29–0.71)	0.52 (0.31–0.72)	0.51 (0.30–0.70)	0.51 (0.31–0.69)

Table 1b	Clinically relevant PCG groups																
	*0 PCGs*	*Cancer*	*Inflam-matory*	*Diabetes*	*Hyper-tension-related*	*Immune*	*Thyroid*	*Pain*	*Mental*	*Asthma*	*Glaucoma*	*HIV/AIDS*	*Heart disease*	*Parkinson*	*Epilepsy*	*Other*	*2+ Diseases*
Overall visits (to specialists; to GPs)	7.9 (3.5; 4.5)	15.8 (9.1; 6.7)	16.3 (8.4; 7.9)	13.5 (5.3; 8.1)	12.2 (4.7; 7.5)	15.1 (8.9; 6.2)	12.7 (5.5; 7.2)	15.1 (6.2; 8.9)	17.5 (8.8; 8.8)	14.5 (6.1; 8.3)	13.2 (6.8; 6.4)	10.5 (5.5; 5.1)	17.4 (6.1; 11.3)	18.5 (6.8; 11.7)	13.4 (5.6; 7.9)	19.7 (8.7; 11.1)	19.6 (9.0; 10.6)
% individuals with one GP (COCI GP = 1)	73.8	71.1	68.8	76.2	77.0	67.9	71.8	73.4	67.9	69.9	74.2	71.4	70.7	69.9	70.8	65.9	70.2
Mean overall COCI (IQR)	0.51 (0.29–0.71)	0.42 (0.23–0.55)	0.44 (0.25–0.58)	0.57 (0.33–0.8)	0.55 (0.33–0.76)	0.47 (0.27–0.62)	0.48 (0.27–0.67)	0.52 (0.30–0.71)	0.52 (0.31–0.71)	0.52 (0.30–71)	0.40 (0.25–0.50)	0.56 (0.33–0.76)	0.56 (0.34–0.77)	0.51 (0.29–0.71)	0.54 (0.33–0.78)	0.55 (0.33–0.76)	0.51 (0.30–0.70)

Table 1c	**Cluster analysis groups (10% sample)**						
	*0 PCGs*	*High-cost high-need*	*Mix of inexpensive PCGs*	*Oldest at high risk*	*One costly PCG*	*Hypertension-related*	*Mental diseases*
Overall visits (to specialists; to GPs)	9.9 (3.9; 6.0)	20.2 (8.6; 11.6)	17.1 (7.3; 9.8)	17.6 (6.2; 11.3)	16.1 (6.7; 9.4)	12.7 (4.4; 8.3)	18.5 (8.9; 9.5)
% individuals with one GP (COCI GP = 1)	73.4	70.4	70.7	67.0	72.0	77.5	68.0
Mean overall COCI (IQR)	0.50 (0.29–0.71)	0.53 (0.30–0.75)	0.48 (0.27–0.64)	0.52 (0.30–0.73)	0.49 (0.3–0.67)	0.55 (0.33–0.75)	0.51 (0.30–0.67)

* based on **N** = **171′646 for COCI overall, and N** = **133′929 for COCI GP**.

A higher morbidity was weakly associated with overall COCI, and the effect was not linear (Fig. 1a): overall COCI among individuals with 1 PCG or, similarly, with 3+ PCGs was 2 percentage points (p.p.) higher than overall COCI among individuals with 0 PCGs.

**Fig. 2:**
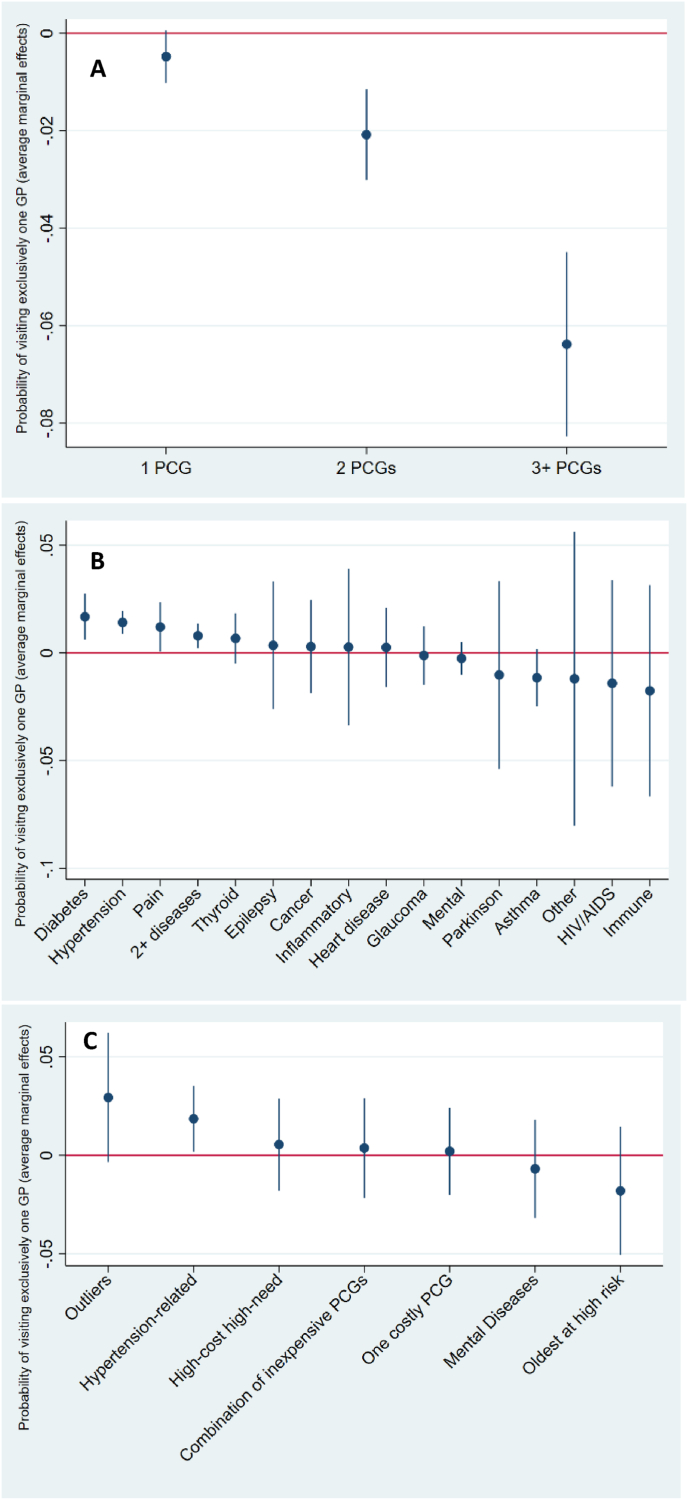
Average marginal effects in percentage points in 2015 of various multimorbidity specifications on probability of visiting exclusively one GP, COCI GP (fully adjusted model) **A)** simple PCG counts approach **B)** clinically relevant disease groups of PCGs (expert-based approach) **C)** cluster analysis (data-driven approach).

The proportion of individuals with one GP (COCI GP = 1.0) was substantially larger among those with 0 PCGs (74%) than among those with 3+ PCGs (66%) (Table 1a). Individuals having 3+ PCGs were 6 p.p. less likely to visit exclusively one GP (Fig. 2a).

The results of all generalized linear models, expressed in average marginal effects with significance levels, underlying Fig. 1, Fig. 2(a-c) can be found in Supplement (Table 3 (a-c)).

### 3.3 Associations between COC and (multi)morbidity expressed via clinical-based approach

Several disease groups showed generally high overall COCI, e.g., Diabetes, Mental and Heart diseases, Asthma, Pain, Hypertension-related diseases (Table 1b).

Individuals suffering from Cancer, Inflammatory or Glaucoma had more visits to the specialists than GPs (Table 1b), and these diseases were the only ones significantly associated with 7 p.p., 5 p.p. and 11 p.p. lower overall COCI, respectively (Fig. 1b).

Among those with any diseases, the Hypertension-related group had the highest proportion of individuals visiting exclusively one GP (77%), and on average visited GPs almost twice more often than specialists (Table 1b). Thus, membership of the Hypertension-related group was the only significant positive association between a disease group and the probability of visiting one GP exclusively (2 p.p.).

### 3.4 Associations between COC and (multi)morbidity expressed via data-driven approach

Members of all clusters had more visits to GPs than to specialists. The members of the “Hypertension-related” cluster had the highest overall COCI (0.55), and had almost twice more visits to GPs than to specialists, 8.3 and 4.4, respectively (Table 1c). Moreover, membership to this cluster came with significantly higher overall COCI and higher probability of visiting exclusively one GP (Fig. 1, Fig. 2c).

The “High-cost high-need” cluster had second highest overall COCI (0.53) (Table 1c). The analysis of associations showed that “High-cost high-need” members were likely to have 4 p.p. higher overall COCI, the strongest association with overall COC among all the other clusters (Fig. 1c).

The members of “Mental health diseases” cluster, together with those of the “Oldest at risk” had the lowest proportion of individuals with exclusively one GP (68% and 67%, respectively) (Table 1c), which was also shown in the analysis of associations (Fig. 2c).

### 3.5 Additional variables associated with COC, relevant in Swiss context

The analyses showed that both COC indices (overall and GP) were significantly and positively related to age, being male, having an insurance contract with gatekeeping, and negatively related to residing in the French-speaking part of Switzerland (Supplement, Tables 3a–3c). A health insurance model with gatekeeping was similarly associated with 2 p.p. higher overall COCI and higher probability of visiting one GP exclusively. A higher level of deductible was associated with 2 p.p. higher probability of visiting one GP exclusively.

All aforementioned models and analyses were reproduced for other visits-based COC indices (UPC, HH and KL) (Bazemore et al., 2018; Jee & Cabana, 2006; Lou, 2000) showing similar results. These latter results can be obtained from the authors upon request.

## 4 Discussion

In the present descriptive study, we investigated associations between COC and multimorbidity, and the effect of other potential determinants of COC relevant in Swiss settings. The findings demonstrated that although the relationship between multimorbidity and overall COC was significant and positive, the magnitude of the association was modest. The approach using clinically relevant disease groups showed the largest variations in COC, while simple PCG counts showed the lowest variations and weakest associations. The data-driven approach revealed that most complex individuals (“high-cost high-need”) tended to have higher overall COCI. Even though gatekeeping is not generalized in Switzerland, we found that 70% of the sample exclusively visited one GP, which is an important proportion for COC in primary care. COC GP was lower for patients with multiple PCGs, for “Oldest at risk”, and patients with Mental diseases. The other significant determinants of COC were age, gender, residing in the French-speaking region of Switzerland, the deductible of the insurance, and the insurance model with gatekeeping.

Our findings demonstrated that multimorbidity, using the PCG counts approach, is significantly but weakly positively associated with COC. This is partially in line with earlier studies, which applied the approaches of chronic condition counts or morbidity indices (Chung et al., 2016; Dreiher et al., 2012; Kohnke & Zielinski, 2017; Ryvicker & Russell, 2018; Sharma et al., 2009). Stemming from existing literature on multimorbidity and fragmentation of care (Pham, Schrag, O'Malley, Wu, & Bach, 2007; Sheaff et al., 2015), one may assume that higher multimorbidity is associated with lower COC. This held true in an earlier study from Taiwan (Wang et al., 2020), and supported by our findings for multimorbidity expressed via simple PCG counts related to COCI GP (probability to visit exclusively one GP). However, for overall COCI we found a positive relationship. Similarly, results based on the cluster approach revealed that the members of the “High-cost high-need” cluster are likely to have higher overall COC, in line with previous literature that showed that sicker patients with more health care needs experienced greater COC (Lei, Intrator, Conwell, Fortinsky, & Cai, 2020). One explanation for the positive relationship refers to the limitations of claims-based data, that may not fully capture all medications, services and consultations needed by most complex patients. Another explanation is related to potential confounding effects with health status, such that high COC index reflects rather health deterioration than continuity of care per se, which was found in an earlier study (DuGoff, Bandeen-Roche, & Anderson, 2016). Thus, for patients with more (severe) morbidities we may observe higher measured COC. And the general population in this study, consisting of 70% individuals without PCGs, may have fewer visits to doctors in general, which results in a larger weight of each single visit to a doctor in COC calculations, diminishing the indices (Dreiher et al., 2012). Finally, our analysis cannot rule out that frequent visits to multiple doctors may simply be appropriate for most complex patients (van Servellen, Fongwa, & Mockus D'Errico, 2006).

All the above raise an important consideration that continuity-improving strategies are important not for all, but for specific multimorbid patients (van Servellen et al., 2006). On the one hand, the relationship between COC and number of PCGs was shown positive, implying that individuals with more multimorbidity experience higher COC. On the other hand, the clinical-based or cluster approach showed substantial variation depending on the type of disease or complexity of healthcare need and use. This variation implies that COC improvement is not universal, and depends on visit patterns and needs of patients with different diseases. For example, for some patients, continuity of a single provider or a team may be unnecessary and impractical to maintain over time. Presumably, a certain threshold of complexity exists, where patients with lower or no complexity have a lower need to be continuously followed-up by the same provider and may visit multiple ones. By contrast, patients with higher complexity need more regular medical attention, thus, they may expect larger benefits from improved COC. Such patients also likely visit specialists, as their conditions demand specialized care in addition to primary care (Gruneir et al., 2016; Sharma et al., 2009). However, the conventional overall COCI index is likely to mask differences between types of specialists, as it pools all doctors together. Therefore, more granular, disease-specific or visit-specific, COC index is warranted to understand whether such patients experience COC with the relevant specialists.

Additionally, measuring the number of PCGs may not perfectly represent the concept of multimorbidity as the patient may be considered complex irrelative of the number of conditions, and potential inaccuracy in estimation of pharmacy data and mapping to a certain conditions cannot be ruled out (Chini et al., 2011; Huber et al., 2013). Therefore, we explored the effect of distinct PCGs-based disease groups and clusters on COC. Individuals with hypertension were more likely to have higher overall COCI and COCI GP. Individuals with cancer, by contrast, experienced lower COC, likely reflecting the need to be followed-up not only by a generalist but also by an oncologist and various specialists. As overall COCI is undifferentiated and pools all the visits irrespective of provider specialty, it may mask beneficial effects, appropriateness of care and care pathways (Blozik E, Bähler C, Näpflin M, & M, 2020). Therefore, the low indicator of overall COCI may not always be the accurate reflection of lower quality/continuity care, and a more thorough study of an effect of each individual disease on COC is warranted.

While focusing on the other significant determinants of COC, whereas the effect of age and gender on COC was already well documented (CDKnight et al., 2009; Geroldinger et al., 2018; Hong et al., 2010; Hussey et al., 2014; Kao & Wu, 2016; Kohnke & Zielinski, 2017), the effect of model with gatekeeping needs explanation. Consistent with several previous studies (Berg, Schafer, Kringos, & Klazinga, 2018; Forrest et al., 1999; Hurley, Gage, & Freund, 1991; Rotar, Van Den; Sommers & Wholey, 2003), gatekeeping was associated with higher COC (overall and GP) demonstrating that in gatekeeping systems patient care was received from fewer sources, as expected, which fostered continuity as measured by COCI index. Thus, wider introduction of gatekeeping plans is important to consider in strategies aimed at improving COC. Another important factor in the Swiss setting are regional differences, as COC was significantly lower in the French-speaking region. Explanations could stem from cultural differences, such as lower trust of French-speaking Swiss population in the decisions of doctors, and higher need for information provision (DuGoff et al., 2016). Alternatively, the spread of integrated team-based initiatives may explain lower measures of COC in French-speaking regions. Specifically, the structure of such teams involves various healthcare professionals to integrate care across team members (van Servellen et al., 2006), which differs from conventional expression of COC based on the relationship between a single provider and a patient.

## 5 Strengths and limitations

Our study has several strengths. First, to our knowledge, this is the first study in Switzerland investigating association between COC and various expressions of multimorbidity based on PCGs in the older general population. Second, claims-based data from over 240 thousand older insured were available from a large health insurance company, covering over 1 million customers in Switzerland. Third, the data contained detailed billing, patient and provider-level information delivering the most recent evidence on COC and multimorbidity in the older Swiss population. Fourth, obtaining data from various regions allowed us exploring cultural differences in COC, which is of particular importance in a highly decentralized system.

However, some limitations of the study need to be acknowledged. The first limitation relates to the nature of claims-based data, whereby we do not have diagnostic information and information about referrals from primary care providers. This is important to acknowledge, as the patient would be assigned a low COC if referred to multiple specialists, but in fact it would not result in lower quality care, as it was appropriate and well-coordinated by a referring physician. It is has been shown that referring a patient maintains COC even though a new provider is introduced in the care chain (Geroldinger et al., 2018).

Second, while the PCGs approach is validated (Chini et al., 2011; Huber et al., 2013; Lamers & van Vliet, 2004), it has certain limitations. For example, morbidity status based on drug data may be inaccurately estimated due to the lack of information on drugs beyond outpatient care only, or those not reimbursed by the mandatory health insurance. Additionally, the simplifying assumption of the PCG approach, that the drug is used exclusively for the treatment of a particular condition at any stage of the disease, is not always fulfilled in practice. We should additionally acknowledge the limitations related to the inability to interpret our found associations as being causal, as the analysis was performed for the single year 2015 and reverse causality and endogeneity bias cannot be excluded. The last limitation relates to the complex nature of COC that cannot be comprehensively captured using claims-based data. Interpersonal or informational continuity are not included in COC measures, indeed. Our longitudinal visit-based continuity measures therefore lack information about quality of interpersonal relationships between a provider and a patient.

## 6 Conclusions and implications

Our findings revealed that overall COC was significantly positively associated with high patient complexity, although COC varied by specific condition. As such, our results trigger recognition of the necessity to improve continuity of care for patients with multiple chronic conditions, who are especially at risk of care fragmentation. Insurance models with gatekeeping may facilitate COC, prompting considerations to investigate broader integrated care opportunities in the country. Our study therefore has important implications for policymakers, health insurance representatives, medical professionals and clinic/hospital managers. Future research should focus on the development of alternative COC measures, integrating qualitative patient information to reflect the complex nature of COC.

## Ethics approval

Ethics approval for this study was waived by The Cantonal Commission for the Ethics of Research on Human Beings (CER-VD, Lausanne, Switzerland), stating that it does not fall within the scope of the Research Involving Human Beings, and does not require authorization from the Ethics committee to be carried out, since it does not concern a human disease, nor the structure and functioning of the human body.

## Ethics statement

All authors have seen and agree with the content of the manuscript. All contributors agreed to submit this paper for publication. Neither author has any conflict of financial or other interest. The authors state that the data and methodology used in the research are proprietary.

This work was supported by the Swiss National Science Foundation (Award number: 407440_183447), within the National Research Programme 74 "Smarter Health Care" (NRP 74). The funding agreement ensured the authors’ independence in designing the study, interpreting the data, writing, and publishing the report.

## Author statement

AN: Formal analysis, Software, Writing- Original draft preparation, Visualization, Investigation, Writing- Reviewing and Editing. IPB: Methodology, Supervision, Validation, Conceptualization. CB: Data curation, Software, Validation, Formal analysis. CP: Conceptualization, Validation, Writing- Reviewing and Editing. JW: Validation, Conceptualization, Methodology, Writing- Reviewing and Editing. JM: Conceptualization, Methodology, Supervision, Writing- Reviewing and Editing, Funding acquisition, Project administration.

## Declaration of competing interest

The authors declared no potential conflicts of interest.

## Footnotes

1 i.e. general internal medicine; allergology and clinical immunology; anesthesiology; angiology; cardiology; surgery; dermatology and venereology; endocrinology and diabetology; gastroenterology; gynecology and obstetrics; physical medicine and rehabilitation; neurology; medical oncology; ophthalmology; otorhinolaryngology; pathology; pulmonology; psychiatry and psychotherapy; radiation oncology and radiotherapy; radiology; rheumatology; and urology.
