# Supplementary material for: Continuity of care and multimorbidity in the 50+ Swiss population: An analysis of claims data
Source: SSM Popul Health. 2022 Mar 9;17:101063. doi: 10.1016/j.ssmph.2022.101063 (PMC8928125; doi:10.1016/j.ssmph.2022.101063)
Supplement: Multimedia component 1 [file mmc1.docx]

# Supplement.

**Table 1.** Grouping of 34 PCGs into disease categories.

| *1. Cancer (N=1’205)* | *Cancer (kre) + Complex Cancer(krk) + Hormone sensitive tumor(kho)* |
| --- | --- |
| *2. Inflammatory (N=387)* | *Crohn's disease/ ulcerative colitis (mcr) + Psoriasis (pso) + Rheumatism (rhe)+* *Disease of the brain or Spinal cord: multiple sclerosis (msk)* |
| *3. Diabetes (N=4’269)* | *Diabetes type 1(dm1)+Diabetes type 2 (dm2)* |
| *4. Hypertension-related (N=20’228)* | *Diabetes with hypertension (dmh)+* *Pulmonary (arterial) hypertension(pah) + High cholesterol (hch)* |
| *5. Immune (N=321)* | *Auto -immune disease (aik)+* *Transplants (tra)* |
| *6. Pain (N=3’398)* | *Chronic pain (smc)+ Neuropathic pain (smn)* |
| *7. Thyroid (N=3’355)* | *Thyroid disorder (thy)* |
| *8. Mental (N=9’064)* | *Depression (dep)+ Bipolar disorder (bsr)+ Addiction excl. nicotine (abh) + Attention deficit hyper- activity disorder (adh)+* *Alzheimer's (alz)+ Psychosis (psy)* |
| *9. Asthma (N=3’358)* | *Asthma (ast) + COPD/Severe asthma(cop)* |
| *10. Parkinson (N=285)* | *Parkinson’s (par)* |
| *11. Epilepsy (N=593)* | *Epilepsy (epi)* |
| *12. Glaucoma (N=3’234)* | *Glaucoma (gla)* |
| *13. HIV/AIDS (N=305)* | *HIV/AIDS (hiv)* |
| *14. Heart disease (N=1’470)* | *Heart disease (car)* |
| *15. Other (N=116)* | *Kidney disease (nie) + Growth disorder (was) + Cystic Fibrosis / Pancreatic Enzymes (zfp) + Diseases of the brain or Spinal cord: other (zns)* |
| *16. No diseases (N=172’924)* | |
| *17. 2+ Diseases (N=15’857)* | |

**
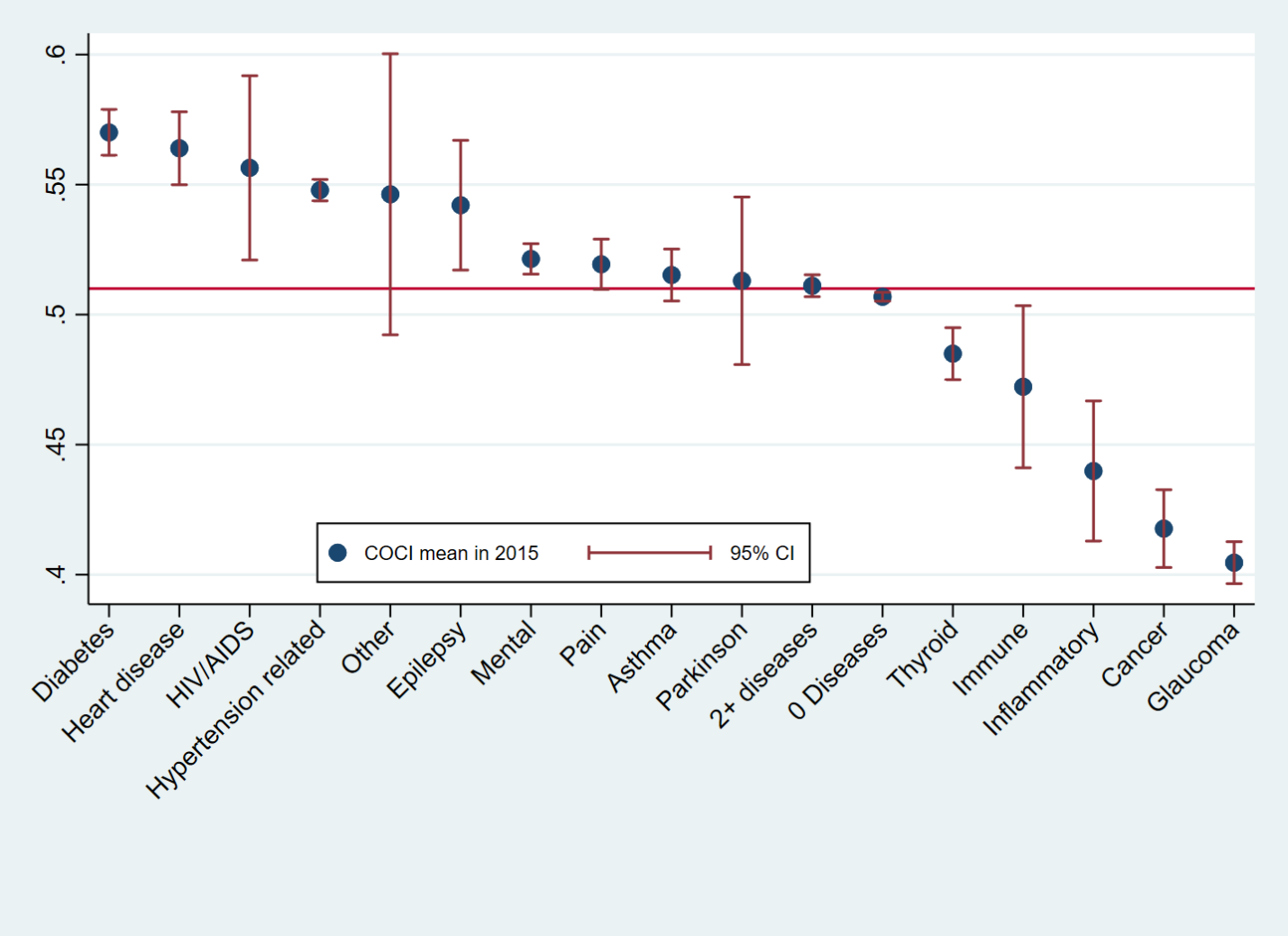
**

**Figure 1.** Mean COCI broad and 95% confidence interval by PCG disease categories in 2015 (sorted)

**Table 2.** Characteristics of the sample

| **Characteristics, total** | **2015** | **2016** | **2017** | **2018** |
| --- | --- | --- | --- | --- |
| N† | 240,419 | 240,321 | 240,231 | 240,089 |
| N deaths |  |  |  | 4,847 (2.02%) |
| Age, years, mean, (SD), med | 63.9 (10.4), 62 |  |  |  |
| Men, n (%) | 115,367 (47.99%) | 115,307 (47.98%) | 115,262 (47.98%) | 115,183 (47.98%) |
| Flag : >3 nights hospital stay, n, % | 22,271 (9.26%) | 24,578 (10.23%) | 27,003 (11.24%) | 29,573 (12.32%) |
| Flag : >5000 chf on medicines, n, % | 7,753 (3.22%) | 9,033 (3.76%) | 10,621 (4.42%) | 12,351 (5.14%) |
| Flag: EMS, n, % | 3,576 (1.49%) | 4,712 (1.96%) | 6,302 (2.62%) | 8,334 (3.47%) |
| Hospital admission (yes), n, % | 31,805 (13.23%) | 33,414 (13.90%) | 35,544 (14.80%) | 38,025 (15.84%) |
| Number of admissions, mean, med, IQR | 0.2,0, 0 | 0.2, 0, 0 | 0.25,0,0 | 0.28,0,0 |
| Length of stay, nights, mean, med, IQR | 2.2, 0,0 | 2.4,0,0 | 2.6,0,0 | 3.1,0,0 |
| Deductible: 300 CHF, n, % | 105,375 (43.83%) | 108,510 (45.15%) | 110,879 (46.15%) | 112,747 (46.96%) |
| Deductible: 500 CHF, n, % | 74,103 (30.82%) | 73,061 (30.40%) | 71,697 (29.85%) | 70,033 (29.17%) |
| Deductible: 1000 CHF, n, % | 12,077 (5.02%) | 11,593 (4.82%) | 11,166 (4.65%) | 9,889 (4.12%) |
| Deductible: 1500 CHF, n, % | 28,823 (11.99%) | 27,068 (11.26%) | 25,284 (10.52%) | 20,709 (8.63%) |
| Deductible: 2000 CHF, n, % | 4,104 (1.71%) | 3,819 (1.59%) | 3,650 (1.52%) | 4,652 (1.94%) |
| Deductible: 2500 CHF, n, % | 15,937 (6.63%) | 16,270 (6.77%) | 17,555 (7.31%) | 22,059 (9.19%) |
| Model with gatekeeping, n, % | 119,917(49.88%) | 122,387 (50.93%) | 126,449 (52.64%) | 131,806 (54.90%) |
| Costs: ambulatory, mean, med, IQR | 5151.9, 2707.0, 1100.6-5780.2 | 5769.3, 2948.9, 1206.9-6370.4 | 6368.4, 3121.6,1263.9-6873.4 | 6537.6, 3204.9, 1301.4-7039.5 |
| Costs: stationary, mean, med, IQR | 1266.7, 0 ,0 | 1393.0,0, 0 | 1533.0, 0,0 | 1758.5,0, 0 |
| Costs: medicines mean, med, IQR | 1523.2,663.9, 203.5-1522.7 | 1694.3,714.5,223.9-1636.1 | 1863.4, 753.9,236.2-1742.9 | 1933.4, 760.1,244.5-1753.4 |
| Costs: participation, mean, med, IQR | 830.7,728.3,486.6-1056.9 | 857.2, 764.2, 505.3-1076.6 | 875.0,789.9, 513.6-1092.1 | 894.6, 807.5, 518.9-1115.7 |
| Total number of annual consultations, mean, med, IQR | 10.0,7,2-14 | 10.6,7,2-15 | 11.0,8,2-15 | 11.8,8,3-17 |
| Number of annual consultations with generalist, mean, med, IQR | 5.6,3,0-8 | 6.0,4,0-9 | 6.1,4,0-9 | 6.5, 4, 0-9 |
| COCI overall, mean, med, IQR | 0.51, 0.46, 0.29-0.71 | 0.51, 0.45, 0.29-0.71 | 0.50, 0.44, 0.29-0.71 | 0.50, 0.44, 0.29-0.71 |
| COCI GP, mean, med, IQR | 0.89, 1, 0.89-1 | 0.89, 1, 0.89-1 | 0.89, 1, 0.85-1 | 0.88, 1, 0.84-1 |
| No PCGs, n, % | 172,974 (71.95%) | 164,219 (68.33%) | 156,379 (65.10%) | 150,992 (62.89%) |
| *Number of specialist doctors visited per year* | 4.73, 4, 3-6 | 4.84, 4, 3-6 | 4.97, 5, 3-6 | 5.10, 5, 3-7 |
| One PCG, n, % | 51,588 (21.46%) | 57,367 (23.87%) | 61,921 (25.78%) | 64,864 (27.02%) |
| *Number of specialist doctors visited per year* | 5.26, 5, 3-7 | 5.29, 5, 3-7 | 5.39, 5,3-7 | 5.52, 5, 4-7 |
| 2+ PCGs, n, % | 15,857 (6.60%) | 18,735 (7.80%) | 21,931 (9.13%) | 24,233 (10.09%) |
| *Number of specialist doctors visited per year* | 5.95, 5, 4-7 | 5.98, 5, 4-7 | 6.09, 6, 4-8 | 6.20, 6, 4-8 |

*† all individuals were observed over 4 years, each year excluding those who moved out of Switzerland*

*Table 3a. Average marginal effects and glm model coefficient estimates for associations between COC and (multi)morbidity expressed via PCG counts in 2015*†

|  | GLM Model estimates | | Average marginal effects | |
| --- | --- | --- | --- | --- |
|  | Overall COCI | COCI GP (=1), visiting one GP | Overall COCI | COCI GP (=1), visiting one GP |
| 0 PCG (ref) |  |  |  |  |
| 1 PCG | 0.06*** | -0.02* | 0.02*** | -0.01* |
| 2 PCGs | 0.05** | -0.06*** | 0.01** | -0.02*** |
| 3+ PCGs | 0.08** | -0.19*** | 0.02** | -0.06*** |
| Age  Age squared | -0.10***  0.00*** | 0.05***  -0.00*** | 0.00*** | 0.00*** |
| Gender (male) | 0.39*** | 0.24*** | 0.10*** | 0.08*** |
| Deductibles 300 (ref) |  |  |  |  |
| Deductibles 500 | -0.02** | 0.06*** | -0.01** | 0.02*** |
| Deductibles >500 | -0.01 | 0.06*** | -0.000 | 0.02*** |
| Health insurance model with gatekeeping | 0.07*** | 0.05*** | 0.02*** | 0.02*** |
| Region Romandie | -0.31*** | -0.05*** | -0.08*** | -0.02*** |

** = p<0.1, ** = p<0.05, *** = p<0.01*

†*Overall COCI – derived from GLM model, COCI GP – derived from probit model.*

*Table 3b. Average marginal effects and glm model coefficient estimates for associations between COC and (multi)morbidity expressed via clinical expert-based approach with relevant PCG disease groups†*

|  | GLM Model estimates | | Average marginal effects | |
| --- | --- | --- | --- | --- |
|  | Overall COCI | COCI GP (=1), visiting one GP | Overall COCI | COCI GP (=1), visiting one GP |
| 0 Chronic condition (ref) |  |  |  |  |
| Cancer | -0.30*** | 0.03 | -0.07*** | -0.02 |
| Inflammatory | -0.20* | 0.03 | -0.05* | -0.04 |
| Diabetes | 0.21*** | 0.19*** | 0.05*** | 0.01 |
| Hypertension related | 0.11*** | 0.16*** | 0.03*** | 0.02*** |
| Immune | -0.08 | -0.17 | -0.02 | -0.05 |
| Thyroid | 0.04 | 0.07 | 0.01 | 0.00 |
| Pain | 0.08** | 0.13*** | 0.02** | 0.00 |
| Mental disease | 0.14*** | -0.03 | 0.03*** | -0.04*** |
| Asthma | 0.03 | -0.12* | 0.01 | -0.04*** |
| Glaucoma | -0.46*** | -0.01 | -0.11*** | 0.00 |
| HIV/AIDS | 0.19 | -0.14 | 0.05 | -0.02 |
| Heart disease | 0.11** | 0.03 | 0.03** | -0.04*** |
| Parkinson | -0.07 | -0.10 | -0.02 | -0.05* |
| Epilepsy | 0.15* | 0.04 | 0.04** | -0.03 |
| Other | 0.02 | -0.12 | 0.05 | -0.07 |
| 2+ Diseases | 0.05*** | 0.09*** | 0.01*** | -0.03*** |
| Age  Age squared | -0.10***  0.00*** | 0.05***  -0.00*** | 0.00*** | 0.00*** |
| Gender (male) | 0.38*** | 0.35*** | 0.09*** | 0.08*** |
| Deductibles 300 (ref) |  |  |  |  |
| Deductibles 500 | -0.02* | 0.04** | -0.01* | 0.02*** |
| Deductibles >500 | -0.00 | -0.00 | 0.00 | 0.02*** |
| Health insurance model with gatekeeping | 0.07*** | 0.08*** | 0.02*** | 0.03*** |
| Region Romandie | -0.31*** | -0.13*** | -0.08*** | -0.02*** |

** = p<0.1, ** = p<0.05, *** = p<0.01*

†*Overall COCI – derived from GLM model, COCI GP – derived from probit model.*

*Table 3c. Average marginal effects and glm model coefficient estimates for associations between COC and (multi)morbidity expressed via data-driven approach with clusters*†

|  | GLM Model estimates | | Average marginal effects | |
| --- | --- | --- | --- | --- |
|  | Overall COCI | COCI GP (=1), visiting one GP | Overall COCI | COCI GP (=1), visiting one GP |
| No chronic condition (ref) |  |  |  |  |
| High-cost high-need | 0.18** | -0.07 | 0.04** | -0.02 |
| Combination of inexpensive PCGs | -0.02 | -0.05 | -0.01 | -0.02 |
| Oldest at high risk | 0.05 | -0.21*** | 0.01 | -0.07*** |
| 1 costly PCG | -0.07 | -0.06 | -0.02 | -0.02 |
| Hypertension-related only | 0.11** | 0.07* | 0.03** | 0.02* |
| Mental only | 0.09 | -0.10* | 0.02 | -0.03* |
| Outliers (mostly Pain) | 0.09 | 0.18** | 0.02 | 0.06** |
| Age  Age squared | -0.10***  0.000*** | 0.03***  -0.000*** | 0.00** | 0.00*** |
| Gender (male) | 0.37*** | 0.24*** | 0.09*** | 0.08*** |
| Deductibles 300 (ref) |  |  |  |  |
| Deductibles 500 | -0.02 | 0.07*** | -0.00 | 0.02*** |
| Deductibles >500 | 0.04 | 0.07** | 0.01 | 0.02** |
| Health insurance model with gatekeeping | 0.06* | 0.06** | 0.02* | 0.02** |
| Region Romandie | -0.30*** | -0.00 | -0.07*** | -0.00 |

** = p<0.1, ** = p<0.05, *** = p<0.01*

†*Broad COCI – derived from GLM model, COCI GP – derived from probit model.*

**Acknowledgements**

This work was supported by the Swiss National Science Foundation (Award number: 407440_183447)
